# “I Teach, Therefore I Am”: The Serial Relationship between Perceived Vulnerability to Disease, Fear of COVID-19, Teacher Identification and Teacher Satisfaction

**DOI:** 10.3390/ijerph182413243

**Published:** 2021-12-15

**Authors:** Anita Padmanabhanunni, Tyrone Pretorius

**Affiliations:** Department of Psychology, University of the Western Cape, Bellville 7530, South Africa; tpretorius@uwc.ac.za

**Keywords:** teaching identification, perceived vulnerability to disease, fear of COVID-19, teaching satisfaction

## Abstract

In early 2020, school closures were implemented globally to curb the spread of the COVID-19 pandemic. In South Africa, emergency remote teaching was not sustainable, and conventional teaching resumed in the context of the second and third waves of the pandemic, heightening fear and anxiety about infection among teachers. The pandemic necessitated shifts in the scope of a teacher’s job, potentially impacting their professional identity and job satisfaction. This study investigated the interrelationship between teaching identification, teaching satisfaction, fear of COVID-19 and perceived vulnerability to disease among a sample of South African school teachers (*n* = 355). A serial mediation analysis supported the hypotheses that teaching identification mediated both the relationship between fear of COVID-19 and teacher satisfaction and the association between perceived vulnerability to disease, fear of COVID-19 and teacher satisfaction. The findings suggest that teacher identification is a potential protective factor, and strengthening professional identification can potentially assist teachers as they negotiate the uncertainty and stress associated with the current pandemic.

## 1. Introduction

Since 2020, in order to curb the spread of COVID-19, governments around the world have implemented a range of physical distancing and other protective measures recommended by the World Health Organization [1,2]. School closure and suspension of face-to-face teaching was one of the most consistently applied strategies globally to mitigate the pandemic, and this strategy necessitated an abrupt shift from traditional classroom-based instruction to emergency remote teaching [3]. Teachers were required to rapidly learn virtual instruction pedagogy and master the use of digital technologies [3,4,5,6]. The challenges associated with remote teaching were aggravated by varying levels of access to digital technology among learners, differing levels of parental involvement and support for online learning and teachers’ competing responsibilities, including home schooling for their own children, caring for elderly or vulnerable family members and balancing domestic and professional responsibilities [7,8]. Even prior to the pandemic, the teaching profession had been identified as a particularly stressful occupation, due to heavy workloads, time-intensive teaching methodologies, limited autonomy, role ambiguity and the need to manage the expectations of administrators and parents [7]. Mandated online teaching, although necessary to promote the continuity of education during the pandemic, has entailed significant shifts in the scope of a teacher’s job, which can potentially impact professional identity and job satisfaction [9].

Professional teacher identity is defined as the extent to which one identifies with being a teacher and includes an individual’s beliefs, values and commitment toward the teaching profession [10]. Teaching satisfaction refers to the level of contentment derived from one’s occupation and is the result of “appraisals of one’s job as achieving or facilitating one’s job values” [11] (p. 172). Researchers have found that job satisfaction in teaching is derived from the gratification of higher-order needs, such as positive teacher-student relationships, supportive collegial relationships and constructive relationships with parents (e.g., [12,13]). These interpersonal relationships constitute important components of teacher identity [13]. Hence, a rapid transition to remote education—which disrupts existing modes of teaching and interpersonal connections with students, colleagues and parents—has the potential to impact teachers’ professional identity and job satisfaction [8]. 

The majority of studies on professional identity and job satisfaction during the pandemic have focused on frontline medical workers (e.g., [14]). These studies have confirmed that access to personal protective equipment, adequate physical health conditions, and formal recognition of work predicted higher levels of job satisfaction and fulfilment (e.g., [15,16]). In contrast, perceived susceptibility to infection and poor working conditions were associated with enhanced fear and anxiety, which negatively impacted job satisfaction and led to frequent absenteeism and professional turnover [14]. Comparatively few studies have focused on teacher job satisfaction and professional identity during the pandemic. [17] For example, a study of university teachers in China found that professional identity negatively predicted job burnout and that job satisfaction had a significant mediating effect on professional identity and burnout. Similarly, [18] a study of rural teachers in western China reported that teacher professional identity played a significant moderating role on job satisfaction. The current study aims to expand the knowledge base in this area by investigating the interrelationship between teaching identification, teaching satisfaction, fear of COVID-19 and perceived vulnerability to disease among a sample of South African school teachers. 

In South Africa, school closures were implemented as part of an initial hard lockdown from March–August 2020 [19]. However, online learning was not sustainable because 90% of South African households do not have internet access [20]. A rotational system of teaching was introduced in August 2020, in which different groups of learners attended school on alternating days [19]. The reopening of schools was reported to have heightened anxiety and worry among teachers about their increased risk of infection [21]. These fears were compounded by reports that approximately 0.6% of teachers (2283 in total) in the country died as a result of contracting the virus during the first and second waves of the pandemic [22]. Overcrowded classrooms, limited access to running water and poor sanitation in many public schools aggravated teachers’ fears of contracting COVID-19 [23]. In addition, children’s difficulties in maintaining social distancing and in consistently using personal protective equipment enhanced teachers’ perceptions of vulnerability to infection [23]. At the time of data collection for the present study (April–July 2021), conventional classroom-based teaching had resumed in the context of the third wave of the pandemic and a slow vaccination rollout [19]. 

The authors speculated that teachers who perceived themselves to be vulnerable to disease would be more fearful of COVID-19 than their peers and that the impact of these two variables on teaching satisfaction would be mediated by the individual’s identification with teaching as a profession. Hence, the following hypotheses were investigated:

**Hypothesis** **1** **(H1).** *Germ aversion would be positively associated with fear of COVID-19.*

**Hypothesis** **2** **(H2).** *Perceived infectability would be positively associated with fear of COVID-19.*

**Hypothesis** **3** **(H3).** *Teaching identification would mediate the relationship between fear of COVID-19 and teaching satisfaction.*

**Hypothesis** **4** **(H4).** *Germ aversion would be associated with heightened fear of COVID-19, which in turn would be associated with lower teaching satisfaction.*

**Hypothesis** **5** **(H5).** *Perceived infectability would be associated with heightened fear of COVID-19, which in turn would be associated with lower teaching satisfaction.*

**Hypothesis** **6** **(H6).** *Teaching identification would mediate the sequential relationship between germ aversion, fear of COVID-19 and teaching satisfaction.*

**Hypothesis** **7** **(H7).** *Teaching identification would mediate the sequential relationship between perceived infectability, fear of COVID-19 and teaching satisfaction.*

Teachers have been positioned as the forgotten frontline workers in the pandemic [24]. They have been found to experience levels of anxiety, depression, post-traumatic stress disorder, burnout and exhaustion [7,25] at rates that are consistent with those reported in large-scale cross-sectional studies focusing on frontline medical workers [26]. Yet there remains comparatively little research on the mental health impacts of COVID-19 on teachers, particularly in developing contexts. This study aims to extend the literature in this area. 

## 2. Materials and Methods

### 2.1. Sample 

The data for this study were collected during April–July 2021. Participants were 355 South African school teachers, most of whom were women (76.6%) and taught at the primary school level (61.1%). Participants had a mean age of 41.9 years (SD = 12.4), and the mean number of years spent in the teaching profession was 15.7 (SD = 11.8). South Africa had around 444,000 schoolteachers in 2019 [27]. Thus, the sample of 355 participants represents a 5.09% margin of error (confidence interval of 95%). Participants from all provinces in South Africa participated in the survey and the majority (82.3%) resided in the Western Cape Province. Participants completed an internet survey created using Google Forms. The survey was distributed via social networking platforms and the institution’s school liaison officer. No incentives were offered for participation in the study. All participants provided informed consent and completed the survey anonymously. Ethical approval for the study was obtained from the University’s ethics committee (ethics code: HS/21/3/8).

### 2.2. Survey Questionnaire 

The first part of the questionnaire investigated participants’ demographic characteristics and consisted of items pertaining to age, gender, area of residence, length of time in the teaching profession and type of school. The second section of the survey consisted of the following four scales:

(a) The Fear of COVID-19 Scale (FCV-19S, [28]) consists of 7 items pertaining to emotional fear reactions to the pandemic. Participants respond on a 5-item Likert scale ranging from 1 (strongly disagree) to 5 (strongly agree). Examples of items include: I am most afraid of coronavirus-19 and I am afraid of losing my life because of coronavirus-19. The scale has demonstrated sound internal consistency reliability, with Cronbach’s alphas ranging from 0.82–0.87 [28,29].
(b) The Perceived Vulnerability to Disease Scale (PVD-Q, [30]) is a 15-item scale that assesses an individual’s perceived vulnerability to infectious disease, specifically perceived infectability (7-item subscale) and germ aversion (8-item subscale). Participants respond to each item on a 7-point Likert scale ranging from 1 (strongly disagree) to 7 (strongly agree). Examples of items include: If an illness is ‘going around’, I will get it (perceived infectability) and It really bothers me when people sneeze without covering their mouths (germ aversion). Sound internal consistency reliability has been reported for the PVD-Q, with Cronbach’s alphas of 0.70, 0.72, and 0.70 for perceived infectability, germ aversion, and total PVDS score, respectively [31].
(c) The Professional Identification Scale (PIS, [32]) is a 10-item measure of professional commitment on a 5-point Likert scale ranging from 1 (*never*) to 5 (*very often*). Examples of items include: I am a person who considers the teaching profession important and I am a person who feels strong ties to the teaching profession. Reliability estimates for the PIS have ranged from 0.71–0.82 [33].
(d) The Teacher Satisfaction Scale (TSS, [11]) is a 5-item scale that asks teachers about their job satisfaction. Responses are measured on a 5-point Likert scale ranging from 1 (strongly disagree) to 5 (strongly agree). Examples of items include: I am satisfied being a teacher and If I chose my career over, I would change almost nothing. The scale has demonstrated sound reliability ranging from 0.77–0.79 [11,34].

### 2.3. Data Analysis

Descriptive statistics, intercorrelations between the variables and reliabilities (alpha and McDonald’s omega) of the scales were obtained using IBM SPSS Statistics for Windows (version 26; IBM Corp., Armonk, NY, USA). Structural equation modelling with IBM SPSS Amos (version 26; IBM Corp.) was used to determine the direct and indirect effects of predictor variables, as well as bootstrapping of confidence levels and *p*-values. In contemporary analysis, confidence intervals are used to determine whether the indirect effects are different from zero. If the confidence interval does not contain zero, the indirect effects are considered significant [35]. 

## 3. Results

The descriptive statistics, intercorrelations and reliabilities for the study variables are reported in Table 1.

All scales, except the Germ Aversion subscale, demonstrated highly satisfactory reliability. The reliability of the Germ Aversion subscale was lower than the standard cutoff of 0.70 but [36] indicated that reliability values above 0.60 may also be considered acceptable.

The two subscales of the PVD-Q, Germ Aversion (r = 0.25, *p* < 0.001) and Perceived Infectability (r = 0.41, *p* < 0.001) were positively related to fear of COVID-19, whereas only the Perceived Infectability subscale was negatively related to teaching identification (r = −0.26, *p* < 0.001) and teaching satisfaction (r = −0.16, *p* = 0.002). Fear of COVID-19 was negatively related to teacher identification (r = −0.12, *p* = 0.028), and teaching identification and teaching satisfaction were positively related (r =0.58, *p* < 0.001).

**Figure 1:**
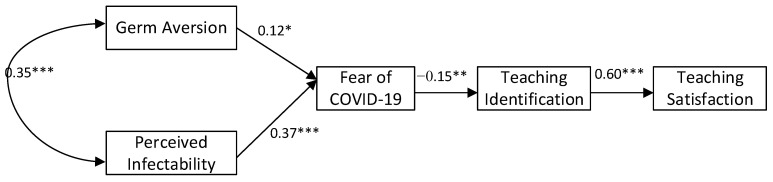
Structural equation model of serial mediation. * *p* < 0.05, ** *p* < 0.01, *** *p* < 0.001.

The structural equation model that was tested is shown in Figure 1 with standardized regression weights.

The results of the structural equation model are reported in Table 2.

The results of the structural equation modelling, reported in Table 2, support all the proposed hypotheses:

1. Germ aversion was positively associated with fear of COVID-19 (β = 0.124, *p* = 0.023).
2. Perceived infectability was positively associated with fear of COVID-19 (β = 0.370, *p* < 0.001).
3. Teaching identification mediated the relationship between fear of COVID-19 and teaching satisfaction (β = −0.087, *p* = 0.005).
4. Germ aversion was associated with fear of COVID-19, which in turn was associated with lower teaching satisfaction (β = 0.019, *p* = 0.030).
5. Perceived infectability was associated with fear of COVID-19, which in turn was associated with lower teaching satisfaction (β = 0.056, *p* = 0.005).
6. Teaching identification mediated the sequential relationship between germ aversion, fear of COVID-19 and teaching satisfaction (β = −0.018, *p* = 0.016).
7. Teaching identification mediated the sequential relationship between perceived infectability, fear of COVID-19 and teaching satisfaction (β = −0.054, *p* = 0.004).

## 4. Discussion

This study aimed to investigate the interrelationship between teaching identification, teaching satisfaction, fear of COVID-19 and perceived vulnerability to disease among a sample of South African school teachers. There were several important findings. First, greater perceived vulnerability to disease (i.e., germ aversion and perceived infectability) was associated with heightened fear of COVID-19 among teachers, which in turn was associated with lower teaching satisfaction. The former finding accords with existing studies involving the general population (e.g., [37]) and frontline health care workers (e.g., [38]). Individuals who perceive their likelihood of infection as high may experience the current pandemic as more life-threatening than their peers and may therefore report heightened fear of COVID-19. For example, [39,40] identified perceived vulnerability to disease (β = 0.96, *p* = 0.000) as a significant predictor of fear of COVID-19 among a sample of Nigerian health care workers. [38] found that fear of COVID-19 was negatively associated with job satisfaction among a sample of Egyptian physicians and positively associated with attrition from the profession. [37] reported that the fear of COVID-19 was significantly associated with depression, anxiety, and stress among a non-clinical Turkish sample. 

The reopening of schools in South Africa in August 2020 required teachers to be in close contact with learners and other teachers. Such proximity can potentially increase the risk of exposure to COVID-19 and may lead to feelings of apprehension about becoming infected or infecting significant others. Perceptions of increased vulnerability to COVID-19 may have been heightened due to the inadequate government supply of personal protective equipment (e.g., masks, hand sanitizers and water tanks) to schools, inconsistent disinfecting of classrooms, poor sanitation and overcrowded classes. Further, media reports of school closures due to infection among teachers and learners [41] may have further aggravated perceptions of vulnerability and fear of COVID-19. Fear is an adaptive response; however, excessive fear can contribute to psychological distress and adversely impact occupational functioning and job satisfaction [17].

Second, the study findings indicate that teacher identification mediated the relationship between fear of COVID-19 and teaching satisfaction. This means that identifying with the teaching profession mitigated the impact of fear of COVID-19 on teaching satisfaction, which suggests that teacher identification is a potential protective factor. Prior research has underscored the mitigating influence of teacher identification on the negative effects of long work hours, large class sizes and dissatisfaction with income status (e.g., [17,18]). These studies have found that teachers with strong professional identities appraise teaching as not merely a job but an avenue to make a difference in children’s lives and contribute to the betterment of society. These teachers also have a strong understanding of their work, accept the demands associated with being a teacher and adjust their expectations according to situational demands [17]. In the context of the current pandemic, it is therefore probable that teachers who identified strongly with their profession would appraise COVID-19 as a significant threat to students’ wellbeing and educational attainment and believe that their work could make a substantial difference to students in exceptional and potentially life-threatening circumstances. These beliefs may have contributed to job satisfaction among teachers with strong professional identities. 

Third, teaching identification was found to mediate the relationship between perceived vulnerability to disease (i.e., germ aversion and perceived infectability), fear of COVID-19 and job satisfaction. This finding lends further support to teacher identification as a potential protective factor. Existing studies of professional identity development [42,43] have proposed that teachers’ identities and emotional responses to external stressors are interrelated. It is therefore probable that teachers who identified with the profession would filter government policies related to school reopening through their own perspective and focus on the aspects of the agenda that were congruent with their own beliefs and ideologies [17]. This approach may influence teachers’ sense of vulnerability to infection, fear of COVID-19 and job satisfaction. For example, appraising the reopening of schools as being in the best interest of students, given the low socioeconomic status of many families and differential access to resources among the population, may align with a teacher’s core value of being an agent of change. The more time teachers spend with high-need students, the more satisfied they may feel, regardless of the challenges involved. This congruence between professional identity and externally imposed policy mandates can influence teachers’ motivation, emotional response to stressors and job satisfaction [42]. 

In the context of the profound negative effects of the pandemic on teacher mental health, the current study makes an important contribution to the literature on resilience resources. It highlights teacher identification as a salient factor in promoting adaptation and coping in the context of the pandemic. This type of information can guide planning interventions and inform policy decisions aimed at enhancing teacher job satisfaction and reducing attrition within the profession. In the South African context, interventions that may prove beneficial in enhancing teacher identification need to be low cost and easy to disseminate. These interventions need to include mental health literacy programs and enhancement of access to mental health resources. Smartphone applications have been found to be beneficial in improving mental health among frontline medical workers and it is probable that these types of interventions could be extended to teachers [24]. 

Existing research on enhancing teacher job satisfaction [44] has highlighted the potential of interventions that focus on generating positive emotion. These studies have drawn on the broaden-and-build theory of positive emotions [45] which proposes that the experience of positive emotion can expand momentary thought-action repertoires and contribute to greater feelings of wellbeing. Examples of these interventions include gratitude journals which entail documenting positive life experiences or “gift of time” which involves contacting a significant other who has made a meaningful contribution to one’s life [46]. Within the context of the pandemic, these types of strategies are cost effective and may serve to activate positive self-appraisals and social support networks. 

The study has certain limitations. First, the use of online data collection may have resulted in teachers with more resources being more likely than their peers to respond to the survey. It would be beneficial for future studies to use different methods of data collection. Second, while teachers from all over South Africa participated in the study, the majority of respondents were predominantly from one province and female, and this skewed sample may impact the generalizability of the findings. Future studies can compensate for this limitation by recruiting a larger and more diverse sample. Finally, the study is cross-sectional in nature and reflects a snapshot of teachers’ experiences during a distinct period of time. Longitudinal research can shed further light on the role of protective factors such as teacher identification in teachers’ responses to COVID-19 and job satisfaction.

## 5. Conclusions

The mental health of teachers during the COVID-19 pandemic has been a largely neglected area of study, with limited research focusing on identifying factors that enhance coping and psychological wellbeing [24]. The study findings underscore the importance of teacher identification in mediating the relationship between fear of COVID-19 and teacher satisfaction and the association between perceived vulnerability to disease, fear of COVID-19 and teacher satisfaction. The findings suggest that when stressful working conditions are coupled with a strong sense of teacher identification, the stressors may be appraised as challenges that can be overcome and therefore may not adversely impact teachers’ job satisfaction. The potential protective role of teacher identification has implications for supporting teachers as they negotiate the uncertainty and stressors associated with the current pandemic. Providing teachers with adequate resources and greater agency to proceed with pandemic-related policy enactments, as well as suitable training to manage COVID-19-related stressors, can enhance their job satisfaction and commitment to the teaching profession.

## Figures and Tables

**Table 1:** Descriptive statistics, intercorrelations and reliabilities for the study variables.

Variable	1	2	3	4	5
1. Germ Aversion	-				
2. Perceived Infectability	0.35 ***	-			
3. Fear of COVID-19	0.25 ***	0.41 ***	-		
4. Teaching Identification	0.08	−0.26 ***	−0.12 *	-	
5. Teaching Satisfaction	−0.02	−0.16 **	0.04	0.58 ***	-
Mean	42.8	28.7	20.9	40.1	17.3
SD	8.4	8.8	7.1	6.9	4.7
Alpha	0.65	0.78	0.91	0.85	0.87
Omega	0.66	0.78	0.91	0.83	0.87

*** *p* < 0.001; ** *p* < 0.01; * *p* < 0.05.

**Table 2:** Direct and indirect effects of the predictor variables and mediator.

Effect	Beta	SE	β	95% CI	*p*
Direct Effects					
1. Germ Aversion → Fear of COVID-19	0.105	0.047	0.124	(0.036, 0.220)	0.023
2. Perceived Infectability → Fear of COVID-19	0.302	0.041	0.370	(0.287, 0.451)	0.001
3. Fear of COVID-19 → Teaching Identification	−0.142	0.053	−0.145	(−0.233, −0.055)	0.006
4. Teaching Identification → Teaching Satisfaction	0.401	0.030	0.600	(0.533, 0.664)	0.001
Indirect Effects					
5. Fear of COVID-19 → Teaching Identification → Teaching Satisfaction	−0.057	0.021	−0.087	(−0.093, −0.022)	0.005
6. Germ Aversion → Fear of COVID-19 → Teaching Satisfaction	0.010	0.005	0.019	(0.009, 0.030)	0.030
7. Perceived Infectability → Fear of COVID-19 → Teaching Satisfaction	0.030	0.009	0.056	(0.013, 0.048)	0.005
8. Germ Aversion → Fear of COVID-19 → Teaching Identification → Teaching Satisfaction	−0.006	0.003	−0.018	(−0.014, −0.002)	0.016
9. Perceived Infectability → Fear of COVID-19 → Teaching Identification → Teaching Satisfaction	−0.017	0.007	−0.054	(−0.030, −0.007)	0.004

## Data Availability

The data presented in this study are openly available in Figshare at DOI:10.25379/uwc.13436252.

## References

[1] Bergeri I., Lewis H.C., Subissi L., Nardone A., Valenciano M., Cheng B., Glonti K., Williams B., Abejirinde I.O., Simniceanu A. (2021). Early epidemiological investigations: World Health Organization UNITY protocols provide a standardized and timely international investigation framework during the COVID-19 pandemic. Influenza Other Respir. Viruses.

[2] World Health Organization (2021). Annex to Infection Prevention and Control during Health Care when Coronavirus Disease (COVID-19) Is Suspected or Confirmed: Interim Guidance, 1 October 2021 (No. WHO/2019-nCoV/IPC/Annex/2021.1).

[3] Hodges C.B., Moore S., Lockee B.B., Trust T., Bond M.A. (2020). The Difference between Emergency Remote Teaching and Online Learning. http://hdl.handle.net/10919/104648.

[4] Kuhfeld M., Soland J., Tarasawa B., Johnson A., Ruzek E., Liu J. (2020). Projecting the potential impact of COVID-19 school closures on academic achievement. Educ. Res..

[5] Moorhouse B.L. (2020). Adaptations to a face-to-face initial teacher education course ‘forced’ online due to the COVID-19 pandemic. J. Educ. Teach..

[6] Whittle C., Tiwari S., Yan S., Williams J. (2020). Emergency remote teaching environment: A conceptual framework for responsive online teaching in crises. Inf. Learn. Sci..

[7] Baker C.N., Peele H., Daniels M., Saybe M., Whalen K., Overstreet S. (2021). Trauma-Informed Schools Learning Collaborative The New Orleans. The Experience of COVID-19 and Its Impact on Teachers’ Mental Health, Coping, and Teaching. Sch. Psychol. Rev..

[8] Kim L.E., Asbury K. (2021). ‘Like a rug had been pulled from under you’: The impact of COVID-19 on teachers in England during the first six weeks of the UK lockdown. Br. J. Educ. Psychol..

[9] Truzoli R., Pirola V., Conte S. (2021). The impact of risk and protective factors on online teaching experience in high school Italian teachers during the COVID-19 pandemic. J. Comput. Assist. Learn..

[10] Richardson P.W., Watt H.M. (2018). Teacher professional identity and career motivation: A lifespan perspective. Research on Teacher Identity.

[11] Ho C.L., Au W.T. (2006). Teaching satisfaction scale: Measuring job satisfaction of teachers. Educ. Psychol. Meas..

[12] Toropova A., Myrberg E., Johansson S. (2021). Teacher job satisfaction: The importance of school working conditions and teacher characteristics. Educ. Rev..

[13] Pepe A., Addimando L., Veronese G. (2017). Measuring teacher job satisfaction: Assessing invariance in the teacher job satisfaction scale (TJSS) across six countries. Eur. J. Psychol..

[14] De los Santos JA A., Labrague L.J. (2021). The impact of fear of COVID-19 on job stress, and turnover intentions of frontline nurses in the community: A cross-sectional study in the Philippines. Traumatology.

[15] Yu X., Zhao Y., Li Y., Hu C., Xu H., Zhao X., Huang J. (2020). Factors Associated With Job Satisfaction of Frontline Medical Staff Fighting Against COVID-19: A Cross-Sectional Study in China. Front. Public Health.

[16] Zhang S.X., Liu J., Jahanshahi A.A., Nawaser K., Yousefi A., Li J., Sun S. (2020). At the height of the storm: Healthcare staff’s health conditions and job satisfaction and their associated predictors during the epidemic peak of COVID-19. Brain Behav. Immun..

[17] Chen H., Liu F., Pang L., Liu F., Fang T., Wen Y., Chen S., Xie Z., Zhang X., Zhao Y. (2020). Are You Tired of Working Amid the Pandemic? The Role of Professional Identity and Job Satisfaction against Job Burnout. Int. J. Environ. Res. Public Health.

[18] Tang Y. (2020). It’s not only work and pay: The moderation role of teachers’ professional identity on their job satisfaction in rural china. Appl. Res. Qual. Life.

[19] Department of Basic Education (2020). Basic Education Gazette for Reopening of Schools.

[20] Mathebula R.N., Runhare T. (2021). Saving the Curriculum or Saving Life? The Risk of Opening Schools in South Africa at the Peak of the Country’s COVID-19 Pandemic. J. Educ. Soc. Res..

[21] Bangani Z. (2020). Teachers’ Fears Are Real and They Need a Willing Ear. New Frame. https://www.newframe.com/teachers-fears-are-real-and-they-need-a-willing-ear/.

[22] Shoba S. (2021). Lessons from Lockdown: South Africa’s Education System Is Just Another Covid-19 Statistic. The Daily Maverick. https://www.dailymaverick.co.za/article/2021-07-18-lessons-from-lockdown-south-africas-education-system-is-just-another-covid-19-statistic/.

[23] Reddy V., Soudien C., Winnaar L. (2020). Disrupted Learning during COVID-19: The Impact of School Closures on Education Outcomes in South Africa. http://repository.hsrc.ac.za/handle/20.500.11910/15402.

[24] Beames J.R., Christensen H., Werner-Seidler A. (2021). School teachers: The forgotten frontline workers of COVID-19. Australas. Psychiatry.

[25] Jakubowski T.D., Sitko-Dominik M.M. (2021). Teachers’ mental health during the first two waves of the COVID-19 pandemic in Poland. PLoS ONE.

[26] Moitra M., Rahman M., Collins P.Y., Gohar F., Weaver M., Kinuthia J., Rössler W., Petersen S., Unutzer J., Saxena S. (2021). Mental health consequences for healthcare workers during the COVID-19 pandemic: A scoping review to draw lessons for LMICs. Front. Psychiatry.

[27] Galal S. (2021). Number of Teachers in Education in South Africa in 2019, by Province. https://www.statista.com/statistics/1262709/number-of-teachers-in-education-in-south-africa-by-province/.

[28] Ahorsu D.K., Lin C.Y., Imani V., Saffari M., Griffiths M.D., Pakpour A.H. (2020). The fear of COVID-19 scale: Development and initial validation. Int. J. Ment. Health Addict..

[29] El-Bardan M.F., Lathabhavan R. (2021). Fear of COVID-19 scale: Psychometric properties, reliability and validity in Egyptian population. Diabetes Metab. Syndr. Clin. Res. Rev..

[30] Duncan L.A., Schaller M., Park J.H. (2009). Perceived vulnerability to disease: Development and validation of a 15-item self-report instrument. Pers. Individ. Differ..

[31] Ahmadzadeh M., Ghamarani A., Samadi M., Shamsi A., Azizollah A. (2013). The investigation of validity and reliability of a scale of perceived vulnerability to disease in Iran. Br. J. Soc. Sci..

[32] Brown R., Condor S., Mathews A., Wade G., Williams J. (1986). Explaining intergroup differentiation in an industrial organization. J. Occup. Psychol..

[33] Veličković V.M., Višnjić A., Jović S., Radulović O., Šargić Č., Mihajlović J., Mladenović J. (2014). Organizational commitment and job satisfaction among nurses in Serbia: A factor analysis. Nurs. Outlook.

[34] Bibi A., Khalid M.A., Hussain A., Saleem H. (2018). Emotional Contagion and job satisfaction among teachers of children with learning disabilities in Pakistan. Eur. J. Spec. Educ. Res..

[35] Kenny D. (2018). Mediation. http://davidakenny.net/.

[36] Van Griethuijsen R.A., van Eijck M.W., Haste H., den Brok P.J., Skinner N.C., Mansour N., Gencer A.S., BouJaoude S. (2015). Global Patterns in Students’ Views of Science and Interest in Science. Res. Sci. Educ..

[37] Satici B., Gocet-Tekin E., Deniz M.E., Satici S.A. (2020). Adaptation of the fear of COVID-19 scale: Its association with psychological distress and life satisfaction in Turkey. Int. J. Ment. Health Addict..

[38] Abd-Ellatif E.E., Anwar M.M., AlJifri A.A., El Dalatony M.M. (2021). Fear of COVID-19 and its Impact on Job Satisfaction and Turnover Intention among Egyptian Physicians. Saf. Health Work.

[39] Boyraz G., Legros D.N., Tigershtrom A. (2020). COVID-19 and traumatic stress: The role of perceived vulnerability, COVID-19-related worries, and social isolation. J. Anxiety Disord..

[40] Osagiator Ariyo J., Olutope Akinnawo E., Chinonye Akpunne B., Oluwasanmi Kumuyi D., Foluke Onisile D. (2021). An Investigation of Associations and Incidence of Anxiety, Depression, Perceived Vulnerability to Diseases, and Fear of COVID-19 among Nigerian Health Care Workers. Arch. Pediatric Infect. Dis..

[41] Evans J. 98 Teachers in Western Cape Positive for Covid-19, as 16 Schools CLose. News24. 2020; 11 June: 1-2. https://www.news24.com/news24/southafrica/news/98-teachers-in-western-capepositive-for-covid-19-as-16-schools-close/.

[42] Deng L., Zhu G., Li G., Xu Z., Rutter A., Rivera H. (2018). Student teachers’ emotions, dilemmas, and professional identity formation amid the teaching practicums. Asia-Pac. Educ. Res..

[43] Zhang Q., Clarke A., Lee C.K.J. (2018). Pre-service Teachers’ Professional Identity Development within the Context of School-Based Learning to Teach: An Exploratory Study in China. Asia-Pac. Educ. Res..

[44] Burić I., Moe A. (2020). What makes teachers enthusiastic: The interplay of positive affect, self-efficacy and job satisfaction. Teach. Teach. Educ..

[45] Fredrickson B.L. (2001). The role of positive emotions in positive psychology: The broaden-and-build theory of positive emotions. Am. Psychol..

[46] Gander F., Proyer R.T., Ruch W., Wyss T. (2013). Strength-based positive interventions: Further evidence for their potential in enhancing well-being and alleviating depression. J. Happiness Stud..

